# Investigating the Effects of Capital Structure and Corporate Governance on Firm Performance: An Analysis of the Sugar Industry

**DOI:** 10.3389/fpsyg.2022.905808

**Published:** 2022-06-29

**Authors:** Akmal Shahzad, Bushra Zulfiqar, Mehmood ul Hassan, Naif Mansour Mathkur, Irfan Ahmed

**Affiliations:** ^1^Department of Business Administration, Preston University, Islamabad, Pakistan; ^2^Pir Mehr Ali Shah Arid Agriculture University, Rawalpindi, Pakistan; ^3^Business Administration Department, Allama Iqbal Open University, Islamabad, Pakistan; ^4^Department of Finance and Banking, College of Business Administration, Jazan University, Jizan, Saudi Arabia

**Keywords:** capital structure, corporate governance, firm performance, Pakistan stock exchange, sugar sector

## Abstract

The objective of this paper is to investigate the impact of capital structure and corporate governance on firm performance. To test the hypothesis of study, data was collected from annual reports of sugar sector companies listed in PSX. This study data covers from 2015 to 2020. The results of study showed that both proxies of capital structure, i.e., D/A and D/E negatively influence the company performance. Whereas two out of three proxies of corporate governance, i.e., board size and chairman/CEO duality negatively indicate association with company performance while audit committee size has a positive impact on the company performance.

## Introduction

Both businesses and the community benefit from good corporate governance (Mubeen et al., 2021a; Aman et al., 2022; Liu et al., 2022). By promoting a sound corporate governance climate, corporate leaders gain the public's confidence (Abbas et al., 2019a, 2021; Ge et al., 2022). Legislative procedures help societies escape risks and problems (Aqeel et al., 2021; Zhou et al., 2021; Rahmat et al., 2022). Several accounting scandals due to malpractices of senior management in business raised the question of corporate governance as well as business integrity (Abbasi et al., 2021a,b,c). It was also considered an important factor for business growth and economic growth as well as the financial market (Aman et al., 2019; Khazaie et al., 2021; Paulson et al., 2021; Su et al., 2021; Yoosefi Lebni et al., 2021). In this way, corporate governance gains a strategic position among the policymaker (Hussain et al., 2017b, 2019, 2021; Mamirkulova et al., 2020; Mubeen et al., 2021a). Datta (2018) highlighted this issue by discussing the effect of corporate governance on organizational efficiency during financial crises.

In a broader term, corporate governance is viewed as a process, customs, policies, and laws, issued by regulating bodies to deal with the daily operations of the corporation (Tricker, 2015). However, corporate governance structure design varies from country to country, depending upon the economic, political, and social context of the country (Hussain et al., 2017b, 2019, 2021; Mamirkulova et al., 2020; Mubeen et al., 2021a). “The integral part of an effective corporate governance regime includes provision for civil or criminal prosecution of individuals who conduct unethical or illegal acts in the name of the enterprise” (Rouse, 2008). In this way it is corporate governance through which both individual and collective interests of shareholders are protected (Abbas et al., 2019c,d; Hussain et al., 2019). Furthermore, the corporate governance is simultaneously responsible to thart the perils leading to financial crises; its smooth and successful operations are aimed at reducing the cost and steering the firm to capital market development (Hussain et al., 2019; Abbas et al., 2020; Abbasi et al., 2021b; Azadi et al., 2021; Wang et al., 2021).

The firm profitability was directly associated with the firm value. The higher a company performs more value it possesses. Coricelli et al. (2011) had carried out their EBRD study of the business firms of Eastern and Central Europe. Contemporarily, Majumdar and Chhibber (1999) studied the negative effect that the level of debt exerted on a company's profitability. It has taken into account the failure of the western corporate governance system in transition regions. Titman and Wessels (1988) and Harris and Raviv (1991) have arrived at different results even regarding the basic facts about the capital structure in their classical empirical studies. The empirical studies on the relationship between the two variables, leverage, and market success, are still insufficient to be decisive, persuasive, and conclusive.

GC (2016) discovered a negative relationship between team size, promoter power, leverage, and profitability. Kumar (2016) discovered a positive relationship between both variables. Similarly, Ahmad and Al-Homaidi (2018) discovered that the “audit community size and board size” had the highest disclosed proxies, while public equity had the smallest disclosure measure for tourism firms.

The managers exploit the eminent domain by expropriating the business assets in achieving their personal goals. They launch such projects which benefit them directly but adversely affect the wealth of the stakeholders (Jensen and Meckling, 1976; Fama and Jensen, 1983; La Porta et al., 1997). For example, the fall of large corporations around the world, such as Enron, Ansett Airlines, Scarf, Retailer Harris, and WorldCom, sparked a global awareness of corporate governance issues. Since the Australian collapses, though, there has been a greater emphasis on disclosures and the integrity of auditors and directors, as well as the need for boards to operate in the best interests of creditors rather than their own. Furthermore, Lockhart and Taitoko (2005) investigated the causes of Ansett Holdings (Ltd. crash)'s and Air New Zealand's biggest corporate defeat, all of which were due to governance weakness in the organization's (stakeholders) interest.

Previous researchers have been only concentrating on evaluating the relationship between corporate governance and corporate performance. However, various researchers employed different methodologies to create the influence of corporate governance on the financial performance of organizations. Kimosop (2011) used regression in his study and found that there was a remarkable link between the board size, non-executives directorship, insider shareholders and board meeting frequency with both ROA and ROE, while Makokha (2014) reviewed a list of variables in his study namely: board size, board composition, leverage and risk committee—how they affect performance and he used content and regression analysis.

With the given background, the objective of this study is to explore the complicated topic of corporate governance—performance. This study takes the case of Pakistan which is a developing country and has quite a different business environment than developed countries studies earlier. More specifically, the objective of this study is

- To examine the relationship of capital structure with firm performance.
- To investigate the relationship of corporate governance with firm performance.

The rest of the article is laid out as follows: Section Literature Review is a review of prior research. Our proposed methodology is outlined in Section Methdology. Section Result and Discussion summarizes our key findings, while Section Conclusion concludes with several key policy recommendations.

## Literature Review

The empirical studies on the relationship between the performance of a business unit and leverage can be categorized into two groups. The leverage is considered as the dependent variable by the one group of researchers. They try to consider it determinants which include indicators of the performance of a company. From the view point of the other group, the leverage is considered as one of the explanatory variables to evaluate the performance of a company. We are shaping this study according to the proponents who consider the leverage as the desired variable for maximizing and enhancing the value of a firm.

Oladeji et al. (2015) examined the relationship between capital structure and firm performance by using 10 years of data for Nigerian companies and concluded the positive and significant relationship between both variables. Whereas Another Malaysian study was conducted by San and Heng (2011) on corporate performance and the capital structure. For this study, Construction Industry was selected for the year 2005–2008. The sample comprised 49 corporations. The relationship between company performance and capital structure was found significant. Similarly, Nawaz and Ahmad (2017) found a significant relationship between short-term, long-term debt, and return on assets. He added that financial performance would be higher if the firm has a lower short- and long-term debt ratio but a number of shareholders and board size were irrelevant to return on assets. While all included variables, e.g., short term, long term debt, number of shareholders, and board size was also insignificant to return on equity. Another study conducted by Tailab (2014) used similar profitability proxies to explore the effect of capital structure on financial performance and found a significant relationship between the total debt and both dependent variables such as ROA and ROE. Whereas firm size (proxy used sale) has a negative relationship with ROE.

On the other hand, Core et al. (1999) observed that the compensation paid to the CEO is lesser when the board chairperson is not the same person as the CEO, they are two separate and independent positions. A negative relationship between auditor independence (taking into consideration the fees concerning non-audit and audit) and earnings management was brought out by Frankel et al. (2002). Mitton (2001) explored that the governance-related variables induced a strong impact on the performance of 398 corporations taken as a sample in 1997 and 1998, the period of the East Asian Crisis. The corporate governance criteria were analyzed for a sample of 398 corporations from 1990 to 1999 in an empirical study conducted by the authors of Wharton and Harvard business schools. Agarwal and Knoeber (1996) investigated the role of various corporate mechanisms and noticed that interdependence existed between these mechanisms. It was deduced that it is not necessary to have a positive performance yielded by a single mechanism. The combination of mechanisms could be more productive. Various factors board composition (Garcia-Meca et al., 2015; Koji et al., 2020), board gender equity (Liu et al., 2014; Mubeen et al., 2020), board independence (Joseph et al., 2014), stock holding of board members (Bhagat and Bolton, 2013), and whether the Chairman and CEO seats are held by the same or two different individuals (Bhagat and Bolton, 2008) were also investigated. Whereas Ali and Irfan (2020) investigated the effect of corporate governance on the share price of listed companies in PSX and found that concentration of ownership did not affect the stock prices.

While Demsetz (1985) concluded that the incentives, monitoring skills, and the objectives could vary for each class of management like individuals, families, corporations, and financial institutions. For the monitoring aspect, the individuals could contribute positively and effectively to a firm's growth as they are wholly and strongly involved in the development of the company. On the other hand, the financial institutions could efficiently monitor the managers by virtue of the skills and resources delegated to them by the firms to secure their interests. Furthermore, Margaritis and Psillaki (2007) found the above relationship to work in small and medium-sized firms in New Zealand which also supported the agency cost theory. For the developing markets, the influence of leverage on the performance of companies was addressed by many studies. Whereas, Muhammad et al. (2014) described the negative relationship of capital structure with a firm performance by using data from Pakistan corporate sector from 2009 to 2013. Whereas Abbas and Awan (2016) observed a low effect of financial leverage and firm size on a key elements of corporate governance using data from 69 manufacturing firms listed in Pakistan stock exchange.

According to Lipton and Lorsch (1992), the performance of a firm is proportional to the board size. Asmaller board is seen to improve the firm performance (Malik et al., 2017; Naveed et al., 2019; Shahzad et al., 2019; Asad et al., 2020a; Ashraf et al., 2020). The larger board has the benefit of maximum monitoring but its aim is undermined by its poor communication and poor decision making (Badar and Irfan, 2018; Abbas et al., 2019b; Ali and Irfan, 2020; Malik et al., 2020; Ali et al., 2021; Zhu et al., 2021). They noticed that board size induces a negative effect on the company's performance (Zhang et al., 2012; Asad et al., 2020b; Yan et al., 2020; Ahmad et al., 2021). Anderson and Mansi (2003) observed that the larger boards are more impressive for the creditors. They invest trust and confidence in such a situation which safeguards their interests as the more effective tool to monitor the financial accounting processes are used in the shape of a larger board. It helps in bringing the cost of the debt down as the creditors feel safer. It was noted by Brown and Caylor (2006) that the large board size of 6–15 members of the firms tends to produce a higher return on the equity and bring forth higher profit margins. It is considered that such large boards would exercise effective and positive control over the business. It has been noticed that the business problems are higher when two positions, CEO and Chairman, are held by the same person. Furthermore, Narwal and Jindal (2015) discussed the corporate governance relationship with firm performance using the data from the Indian textile sector from 2010 to 2014 and concluded that board size, non-executive directors, and board meeting has no effect on the firm profitability. Whereas director's compensations affect positively the firm profitability while the audit committee members have a negative relationship with firm profitability. Similarly, the long-term debt has a significant relationship with NPR & ROA. While audit committee and board size insignificant association with financial performance concluded by Jamal and Mahmood (2018) while conduction study in Pakistan 2007–2016. Vo and Nguyen (2014) conducted using data from firm listed on Ho stock exchange and determined that board independence, CEO duality, and firm performance has a negative association. Whereas board size has no effect on firm performance. Another similar study conducted by Habib (2016) used data from the Dhaka stock exchange and found a negative relationship between board size and firm performance whereas female members on the board, CEO duality, and board compensation affect firm performance significantly and positively. The aim of Ahmed et al. (2019) is to look into the impact of corporate governance and capital structure on the success of a company. The findings reveal that board size has a favorable association with profitability, although the audit committee has a negative relationship. Furthermore, the current ratio and performance have a positive relationship, while the debt to equity ratio has a negative relationship. PeiZhi and Ramazan (2020) add to the existing literature by demonstrating that the type of governance system that is built on diverse expert stakeholders and a capital structure with a high amount of debt is critical to the overall success of companies. The results show that companies with a board of management, an independent auditor, institutional investors, an audit committee, and female directors do well. Furthermore, we discover that while the leverage ratio increases accounting efficiency, it has a negative effect on public company share prices. In short, the researchers used different proxies to examine the firm profitability, (e.g., AL-Omar and AL-Mutairi, 2015; Menicucci and Paolucci, 2016; Almaqtari et al., 2018; Darayseh and Chazi, 2018; Zheng et al., 2018; Al-Homaidi et al., 2019) used ROA as a first indicator. Whereas Dzingai and Fakoya (2017) used ROE as a second indicator. Another indicator EPS adopted by Waleed et al. (2016) in contrast to prior proxies. In this study, the researchers link corporate governance and profitability literature in Pakistan. Also, this study contributed to the existing literature by using panel data from listed companies in PSX and using different corporate governance indicators.

### Theoretical Framework

The following theoretical framework was developed for this study on the base of an extensive literature review. This model shows the relationship between capital structure, corporate governance, and firm performance.

### Hypotheses

The following hypotheses have been established based on previous literature:

- H_1_: Capital structure has positively associated with firm performance.
- H_1a_: Debt to assets has positively affect firm performance.
- H_1b_: Debt to equity positively influenced the firm performance.
- H_2_: Corporate governance has a significant relationship with firm performance.
- H_2a_: Board size has positive impact on firm performance.
- H_2b_: Chairman duality a positively associated with firm performance.
- H_2c_: Audit committee size has a significant impact on firm performance.

## Methodology

### Research Design

Researchers are being positivist during this study, therefore, adopted the deductive, quantitative approach which is easy to explain based on data analysis results as well as easy to conclude. It is in line with the literature (Anjum et al., 2017; Hussain et al., 2017a; Maqsood et al., 2021) operational definition of each variable described in Table 1.

**Table 1:** Variables and description.

**S. no**.	**Variable**	**Label**	**Description**
1.	Firm performance (Return on Assets)	ROA	A measure of a company's profitability in relation to its overall assets. Profit to the total asset was adopted as a proxy of firm performance
2.	Capital structure (Debt to assets)	DTA	The use of debt finance to purchase new assets is represented by this ratio. Total debt to the total asset was used to calculate this ratio
3.	Capital structure (Debt to equity)	DTE	The use of debt capital to obtain new equity is represented by this ratio. Total debt to total equity was used to calculate this ratio
4.	Board size	BS	This represents the number of board members
5.	Chairman duality	CD	This is an indicator that show how many chairmen held the CEO office. It was coded 1 if chairman held CEO office otherwise 0
6.	Audit committee Size	ACS	Nominated by the board to oversee financial statements and transparency. This presents the number of committee members

### Population, Sample, Variables, and Data

The population is all sugar industry firms listed on Pakistan Stock Exchange. Five firms are studied as sample firms. This study followed the scientific procedures for data collection, which is in line with the past literature (Aqeel et al., 2021, Aqeel et al., 2022; Farzadfar et al., 2022; Li et al., 2022; Yao et al., 2022). Secondary data were collected for 5 years from 2015 to 2020 from companies' annual statements. The convenience sampling approach is used for sample selection. Kouloukoui (2019) considered 5 years time frame enough for results robustness.

### Econometric Model

To investigate the relationship between independent variables and dependent variables following regression model was proposed as equation:


$$\begin{array}{r} ROA_{i,t}\;=\;\beta_{1}+\;\beta_{2}\left(\frac{TD_{i,t}}{TA_{i,t}}\right)+\beta_{3}\left(\frac{TD_{i,t}}{TE_{i,t}}\right)+\beta_{4}BS_{i,t} \end{array}$$

(1) $$\begin{array}{r} +\beta_{5}CD_{i,t}+\beta_{6}AC_{i,t}+\varepsilon \end{array}$$

Subscripts *i, t* show firm and year, respectively. Rests of variables are as follow:

ROA = return on assets

TD = total debt

TA = total assets

TE = total equity

BS = board size

CD = CEO duality

AC = audit committee size

ε = error term.

## Result and Discussion

### Descriptive Statistics

Results in Table 2 reveals that the mean of the return on asset is about −0.65% which indicates that every Rs. 100 value of assets of the sampled firm shows a loss of Rs. 0.65. The average board size of the sampled firm is 7. Results also indicate that 34% of the firm have separate persons occupying the position of CEO and Board chair. The average number of the member in the Audit Committee of the sampled firm is 3. The mMean debt to equity and debt to assets ratio is 251.29 and 97.20%, respectively, which states that the company debt level is too high and the company is at high level of risk. A total of 97.20% of the company assets have been financed through debt which was also the reason of loss on ROA.

**Table 2:** Five year summary of descriptive statistics.

	**ROA**	**D/A**	**D/E**	**BS**	**CD**	**AC**
Mean	−0.0065	0.9720	2.5129	7.8522	0.3391	3.0870
Standard Error	0.0173	0.1405	0.3836	0.0910	0.0443	0.0264
Median	0.0184	0.6918	1.9649	8.0000	0.0000	3.0000
Standard Deviation	0.1854	1.5071	4.1141	0.9755	0.4755	0.2830
Range	1.5739	12.0389	34.5279	3.0000	1.0000	1.0000
Minimum	−1.2071	0.1241	−1.9513	7.0000	0.0000	3.0000
Maximum	0.3669	12.1630	32.5766	10.0000	1.0000	4.0000
Sum	−0.7471	111.7776	288.9851	903.0000	39.0000	355.0000
Count	115	115	115	115	115	115

### Correlation Analysis

Correlation is a statistical approach that shows a relationship in two or more variables and these variables fluctuate together over a period. A positive correlation indicates that variables increase or decrease in parallel. A negative correlation indicates the extent to which one variable increases as the other decreases.

Table 3 shows that return on assets is negatively correlated with debt to equity ratio (−0.040), debt to assets ratio (−0.704), board size (−0.086), and CEO duality (−0.274) at a 5% level. Also, there is a positive correlation between return on assets and the number of members on the audit committee. The result indicates that if a firm has a high debt level and high finance, their assets and then its return on assets will be decrease and vice versa. Board size and CEO duality also affect their return on assets because the larger board and CEO duality lead to a decrease in the income and return on assets of the company.

**Table 3:** Correlation.

	**ROA**	**D/A**	**D/E**	**BS**	**CD**	**AC**
ROA	1.000					
D/A	−0.704	1.000				
D/E	−0.040	−0.147	1.000			
BS	−0.086	0.108	−0.262	1.000		
CD	−0.274	0.345	0.062	−0.175	1.000	
AC	0.088	−0.080	0.034	0.015	−0.156	1.000

### Regression Analysis

The adjusted *R* square value is 49.9% which shows that the independent variable is account for a 49.9% variation in the dependent variable.

### Regression Equation

The results in table 4 reveals that return on asset = −0.0874 (D/A) −0.0071 (D/E) −0.0106 (BS) +0.0220(AC) −0.0094 (CD) + 0.1146. The *t*-value of debt to equity ratio and debt to asset ratio −2.275 and −9.79, respectively are greater than 2.00 so the result is significant and both variables negatively affect the firm performance. Furthermore, *p*-value of D/E and D/A was 0.025 and 0.0000, respectively, which are less than 0.05 (level of significance) which states that capital structure is significant and negatively affects the firm performance.

**Table 4:** Regression Result.

	**Coefficients**	**Standard error**	**t stat**	***p*-value**
Intercept	0.1146	0.1750	0.6546	0.5141
D/E	−0.0071	0.0031	−2.2753	0.0248
D/A	−0.0874	0.0089	−9.7901	0.0000
BS	−0.0106	0.0134	−0.7897	0.4314
AC	0.0220	0.0440	0.4994	0.6185
CD	−0.0094	0.0286	−0.3270	0.7443
R	0.722		R^2^	0521
Adjusted *R*^2^	0.499		Standard Error	0.131

The *t*-value of board size, CEO duality, and audit committee are −0.79, −0.327, and 0.499, respectively which are less than 2.0 so that result is not significant. Furthermore, *p-*value of all three-dependent variables, i.e., the board size, CEO duality, and audit committee is >0.05, which shows that the independent variable (Corporate Governance) does not significantly affect the dependent variable (Firm Performance).

## Conclusion

We have attempted to explore the relationship among the capital structure, performance of the business, and corporate governance in the 23 business units of the sugar industry of Pakistan which are on the list of the Pakistan Stock Exchange during 2015–2020. We use firm performance as a dependent variable and corporate governance as well as capital structure as an independent variable.

Results report that debt to equity ratio, total debt to assets ratio, board size, and chairman duality have a negative and significant impacts on the performance of the firm. The performance of the firm will increase with a decrease in board size and debt to assets ratio and debt to equity ratio. On the other hand, the audit committee has a positive relationship with companies performance, which states that if the companies increase their audit committee members then it will lead to an increase in the performance of the company.

## Data Availability Statement

The raw data supporting the conclusions of this article will be made available by the authors, without undue reservation.

## Author Contributions

All authors listed have made a substantial, direct, and intellectual contribution to the work and approved it for publication.

## Conflict of Interest

The authors declare that the research was conducted in the absence of any commercial or financial relationships that could be construed as a potential conflict of interest.

## Publisher's Note

All claims expressed in this article are solely those of the authors and do not necessarily represent those of their affiliated organizations, or those of the publisher, the editors and the reviewers. Any product that may be evaluated in this article, or claim that may be made by its manufacturer, is not guaranteed or endorsed by the publisher.
